# Loss-of-function Mutation in *PMVK* Causes Autosomal Dominant Disseminated Superficial Porokeratosis

**DOI:** 10.1038/srep24226

**Published:** 2016-04-07

**Authors:** Jiuxiang Wang, Ying Liu, Fei Liu, Changzheng Huang, Shanshan Han, Yuexia Lv, Chun-Jie Liu, Su Zhang, Yayun Qin, Lei Ling, Meng Gao, Shanshan Yu, Chang Li, Mi Huang, Shengjie Liao, Xuebin Hu, Zhaojing Lu, Xiliang Liu, Tao Jiang, Zhaohui Tang, Huiping Zhang, An-Yuan Guo, Mugen Liu

**Affiliations:** 1 Key Laboratory of Molecular Biophysics of Ministry of Education, Department of Genetics and Developmental Biology, College of Life Science and Technology, Huazhong University of Science and Technology, Wuhan, Hubei 430074, PR China; 2 Department of Dermatology, Union Hospital, Tongji Medical College, Huazhong University of Science and Technology, Wuhan, Hubei, 430022, PR China; 3 Department of Bioinformatics and Systems Biology, College of Life Science and Technology, Huazhong University of Science and Technology, Wuhan, Hubei, 430074, PR China; 4 Hubei Polytechnic Institute, Xiaogan, 432000, PR China; 5 Department of Dermatology, Chibi People’s Hospital, Hubei, 537300, PR China; 6 Division of Human Genetics, Department of Psychiatry, Yale University School of Medicine, New Haven, Connecticut 06511, USA

## Abstract

Disseminated superficial porokeratosis (DSP) is a rare keratinization disorder of the epidermis. It is characterized by keratotic lesions with an atrophic center encircled by a prominent peripheral ridge. We investigated the genetic basis of DSP in two five-generation Chinese families with members diagnosed with DSP. By whole-exome sequencing, we sequencing identified a nonsense variation c.412C > T (p.Arg138*) in the phosphomevalonate kinase gene (*PMVK*), which encodes a cytoplasmic enzyme catalyzing the conversion of mevalonate 5-phosphate to mevalonate 5-diphosphate in the mevalonate pathway. By co-segregation and haplotype analyses as well as exclusion testing of 500 normal control subjects, we demonstrated that this genetic variant was involved in the development of DSP in both families. We obtained further evidence from studies using HaCaT cells as models that this variant disturbed subcellular localization, expression and solubility of PMVK. We also observed apparent apoptosis in and under the cornoid lamella of PMVK-deficient lesional tissues, with incomplete differentiation of keratinocytes. Our findings suggest that *PMVK* is a potential novel gene involved in the pathogenesis of DSP and PMVK deficiency or abnormal keratinocyte apoptosis could lead to porokeratosis.

Disseminated superficial porokeratosis (DSP) is the secondly reported clinical subtype of porokeratosis. Subjects, with early onset (usually 5 to 10 years of age) had multiple small keratotic lesions located in both sun-exposed and -unexposed areas, including trunk, genitalia, palm, and toes^1^. Keratotic lesions are featured by an atrophic center rimmed by a prominent peripheral ridge. Histologically, the presence of a cornoid lamella with a thin or absent granular layer and often a thin epidermis is the diagnostic hallmark of porokeratosis. The cornoid lamella is a column of closely stacked parakeratotic cells, which arise from an expansion of abnormal keratinocytes.

Porokeratosis is classified into several subtypes: classic porokeratosis of Mibelli (PM), DSP, disseminated superficial actinic porokeratosis (DSAP), porokeratosis palmoplantariset disseminated (PPPD), porokeratosis punctata palmaris etplantaris (PPPP), and linear porokeratosis (LP). Although porokeratosis was first described more than one hundred years ago, its etiology and pathogenesis are still not fully understood. The mevalonate kinase gene (*MVK*) (MIM: 251170)^2^ and the solute carrier family 17 (vesicular nucleotide transporter), member 9 gene (*SLC17A9*) (MIM: 612107)^2^ have been identified to be the causal genes for DSAP^3^,^4^ and PM^5^. Two linkage loci have been mapped to chromosome 18p11.3 and 12q21.2-24.21, respectively, in two Chinese families with DSP affected members^6^,^7^.

In the present study, we performed exome sequencing and genetic analyses in two large extended Chinese families with members affected with DSP and identified a heterozygous nonsense variation c.412C > T (p.Arg138^*^) in *PMVK* in affected patients. Our functional study showed that this *PMVK* mutation disturbed the subcellular localization, expression and solubility of PMVK. We also observed apoptosis and incompletely differentiated keratinocytes in PMVK-deficient lesional tissues. These findings could enhance our understanding of the *PMVK*–deficient pathogenesis of DSP and the function of *PMVK*.

## Results

### Clinical findings of two DSP families

**Figure 1:**
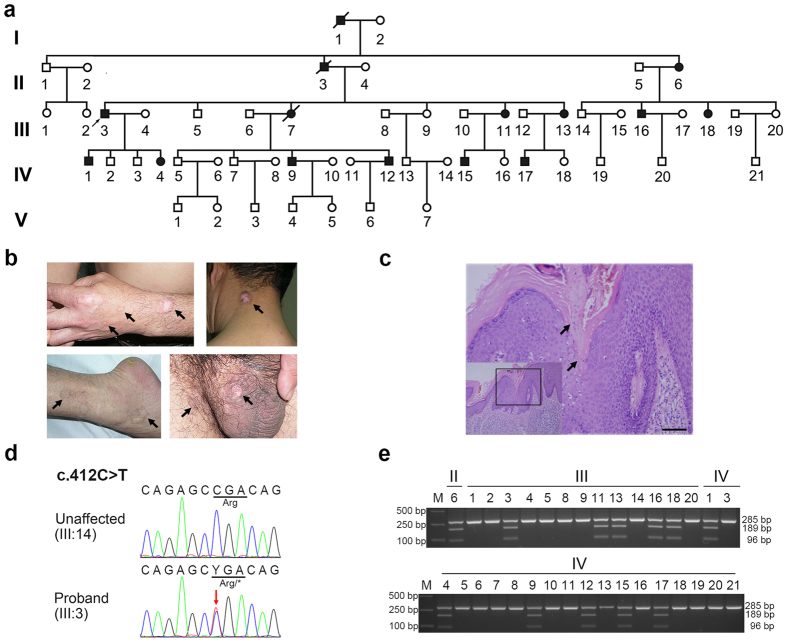
The pedigree tree, clinical manifestations, and mutation information of Family 1. (**a**) Pedigree of Family 1. Black symbols, affected individuals; White symbols, unaffected individuals; Arrow, the proband of the family. Genomic DNA of II:6 (affected), III:14 (unaffected), and IV:1 (affected) were used for whole-exome sequencing. (**b**) Representative keratotic lesions of the skin. Lesions are indicated by arrows on the hand, neck, leg, and scrotum of the proband. (**c**) Histological examination of the lesional tissue from the proband. Typical cornoid lamellae with the absence of a granular layer below the parakeratotic column were indicated by arrows. Scale bar, 50 μm. (**d**) Sanger sequencing of the heterozygous c.412C > T *PMVK* mutation in the proband (III:3). The mutant base is marked by an arrow. The affected codon is underlined. (**e**) Segregation analysis of the mutation in Family 1. The mutation eliminated an Alu I site and led to two additional bands in the restriction fragment length polymorphism (RFLP) analysis.

We characterized two large five-generation families [one from Hubei Province (Family 1; Fig. 1a) and another from Henan Province (Family 2; Supplementary Figure 1a) of China] with members affected with autosomal dominant DSP. The proband (III: 3) in Family1 was a 58-year-old male. The lesions initially appeared when he was 15 years of age, and annular keratotic lesions were observed on his trunk, limbs, feet, buttock, neck, genitalia and perianal area and shown in both sun-exposed and -unexposed areas of his skin (Fig. 1, Table 1). He reported that he and his eldest son as well as the only daughter (IV:1, IV:4) experienced an exacerbation of cutaneous manifestations during autumn months. Other affected individuals in the family showed similar symptoms mainly in sun-unexposed areas of the skin (Table 1). In Family 2, DSP manifested in members when they were 5 to 10 years old and DSP symptoms aggravated around age 16, usually showing a pattern of recurrent episodes and experiencing exacerbations in spring months. The lesions were located in both sun-exposed and -unexposed areas, such as limbs, neck, toes, and palms (Supplementary Figure 1b, Table 1). Most patients in Family 2 experienced pruritus occasionally. More remarkably, subject IV:4 (29-year-old) of Family 2 had hypertrophic plaques at the lesional regions of the forearm and the palm (Supplementary Figure 1c, right top panel). H&E (hematoxylin and eosin) staining of the lesional tissues revealed the presence of cornoid lamellae extending through the stratum corneum with loss of the granular cell layer (Fig. 1c and Supplementary Figure 1c). No family members reported exacerbations of DSP after exposure to the sun. Based on the cutaneous and histopathological examination results, 12 individuals in Family 1 and 11 individuals in Family 2 were diagnosed as having DSP.

### Exome sequencing revealed DSP-associated mutations in *PMVK*

We first performed Sanger sequencing of coding regions and exon-intron boundaries of *MVK* (NG_007702.1) and *SLC17A9* (NG_041785.1) using DNA of two patients (II:6, IV:1) in Family 1. No DSP-associated genetic variants were identified. We then performed whole-exome sequencing of DNAs from the two affected individuals and one unaffected individual (III:14) in Family 1 (Fig. 1a). About 2.7 G bases (per sample) mapped to target exome regions with a mean depth of 52x and a mean coverage of 96.97% of the exomes for at least 10x were generated. The program GATK^8^ was used to perform SNP and InDel discovery and genotype scoring. The identified variants were annotated by ANNOVAR, filtered using the dbSNP137 database^9^ and the 1000 Genomes Project data^10^. We selected ~300 unreported candidate variations (nonsynonymous, splicing, and InDel) from nearly 75,000 genetic variants for each individual. Among the selected candidate variants, 19 were shared by the two affected individuals but absent in the unaffected individual (Supplementary Table 1). Further Sanger sequencing and testing for segregation distortion ruled out all variants except one heterozygous nonsense mutation c.412C > T (p.Arg138^*^) located in *PMVK* (NM_006556.3). These results suggested that *PMVK* might be the pathogenic gene for DSP.

### Implication of *PMVK* nonsense mutation c.412C > T in DSP

Sanger sequencing, restriction fragment length polymorphism (RFLP) analysis and haplotype analysis were performed in Family 1. Variant c.412C > T showed co-segregation with DSP phenotypes (Fig. 1d,e, Supplementary Figure 2 and Supplementary Table 2). However, it was not present in the 500 healthy control subjects. Additionally, we sequenced the exons and exon-intron boundaries of *PMVK* in all members of Family 2, and identified the same c.412C > T mutation (Supplementary Figure 1d and Supplementary Table 2). Variant c.412C > T also co-segregated with DSP phenotypes in Family 2 by the RFLP analysis (Supplementary Figure 1d). This finding also suggested that *PMVK* could be a causal gene for DSP.

### The R138^*^mutation disturbs the cellular localization of PMVK

*PMVK* is located at 1q21.3, and it contains five exons and encodes a 192-amino acid protein, which is expressed in many tissues including epidermal cells in human skin. PMVK, which belongs to the nucleoside monophosphate kinase family, converts mevalonate 5-phosphate to mevalonate 5-diphosphate in the mevalonate pathway, following the biochemical reaction catalyzed by the MVK kinase^11^, which is encoded by MVK, a causal gene already known to be associated with DSAP^3^ and PM^5^.

**Figure 2:**
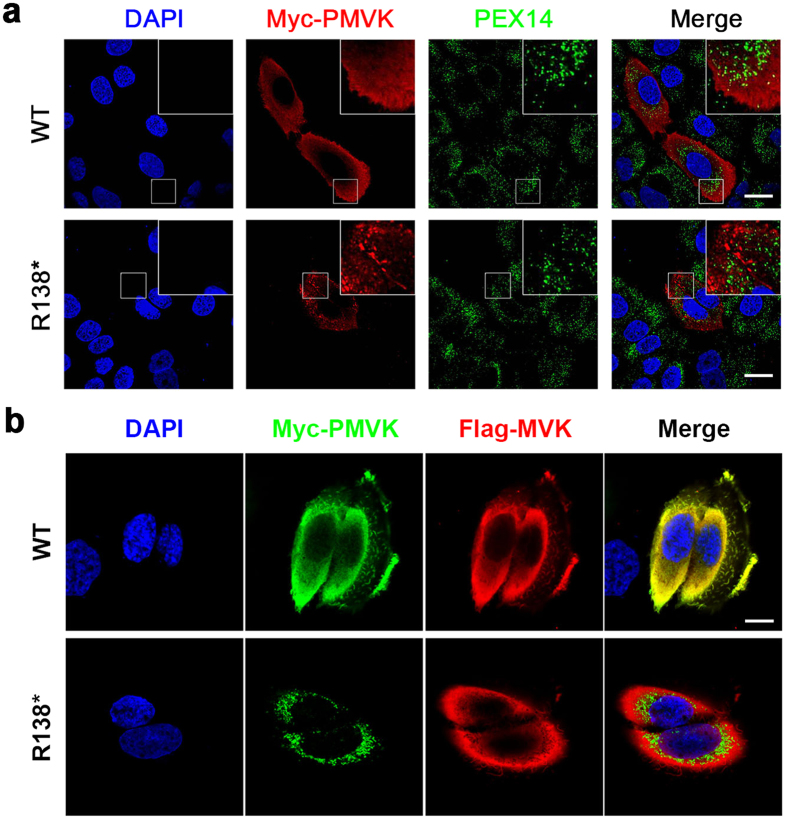
The R138* Mutation Disturbs the Cellular Localization of PMVK. (**a**) Abnormal localization of the R138* mutant PMVK in HaCaT cells. WT PMVK shows a diffuse cytoplasmic distribution, whereas mutant PMVK aggregates in the cytoplasm in a punctate pattern. Neither the WT nor the mutant PMVK co-localizes with the peroxisomal maker PEX14. Boxes on the right top show the partial enlarged details. Green, PEX14; Red, PMVK; Scale bars, 20 μm. (**b**) The R138* mutation destroys the co-localization of PMVK and MVK in HaCaT cells. WT PMVK showed the same dispersed distribution as MVK in cytoplasm, yet mutant PMVK did not co-localize with MVK. Green, PMVK; Red, MVK; Scale bar, 10 μm.

PMVK was initially reported to be localized to peroxisomes through its C-terminal peroxisomal targeting signal (S190-R191-L192)^12^,^13^. Subsequent studies revealed a cytosolic localization of PMVK^14^. To investigate whether the R138^*^ mutation would affect the subcellular localization of PMVK, we transiently expressed Myc-tagged wild or mutant type of human PMVK in HaCaT cells (a keratinocyte cell line derived from the skin cells of an adult human subject). The anti-c-Myc antibody and the anti-PEX14 antibody were used to visualize PMVK and peroxisomes *via* double immunofluorescent staining and confocal microscopy. The wild-type PMVK exhibited dispersed cytoplasmic localization and showed little co-localization with the peroxisomal marker PEX14 (Fig. 2a, upper panel), while the R138* form of PMVK showed punctate localization in the cytoplasm and also did not co-localize with PEX14 (Fig. 2a, lower panel). In addition, co-expression of Myc-tagged wild-type PMVK and Flag-tagged MVK showed the same cytoplasmic distribution in HaCaT cells (Fig. 2b, upper panel), as revealed by anti-Myc and anti-Flag antibodies. As expected, mutant PMVK showed a punctiform distribution around the nuclei and did not co-localize with MVK (Fig. 2b, lower panel). These results suggested that PMVK and MVK were predominantly cytosolically localized, consistent with their closely related roles in the mevalonate pathway. Furthermore, the R138* mutation altered the subcellular localization of PMVK, thus influencing the function of PMVK.

### PMVK with the R138^*^ mutant showed reduced expression and solubility

The amino acid Arg141 located at the C-terminal of PMVK has been reported to contribute to ATP binding^15^. The R138^*^ mutant PMVK lacks the last 55 residues including Arg141. We expressed GST-tagged WT and mutant PMVK in BL21 *E. coli* using the pGEX vector following previous studies^16^. We found that the mutant PMVK formed inclusion bodies, whereas the wild-type PMVK was largely soluble under the same induction condition (Supplementary Figure 3). These results suggest that the R138* mutation could reduce the solubility of PMVK.

**Figure 3:**
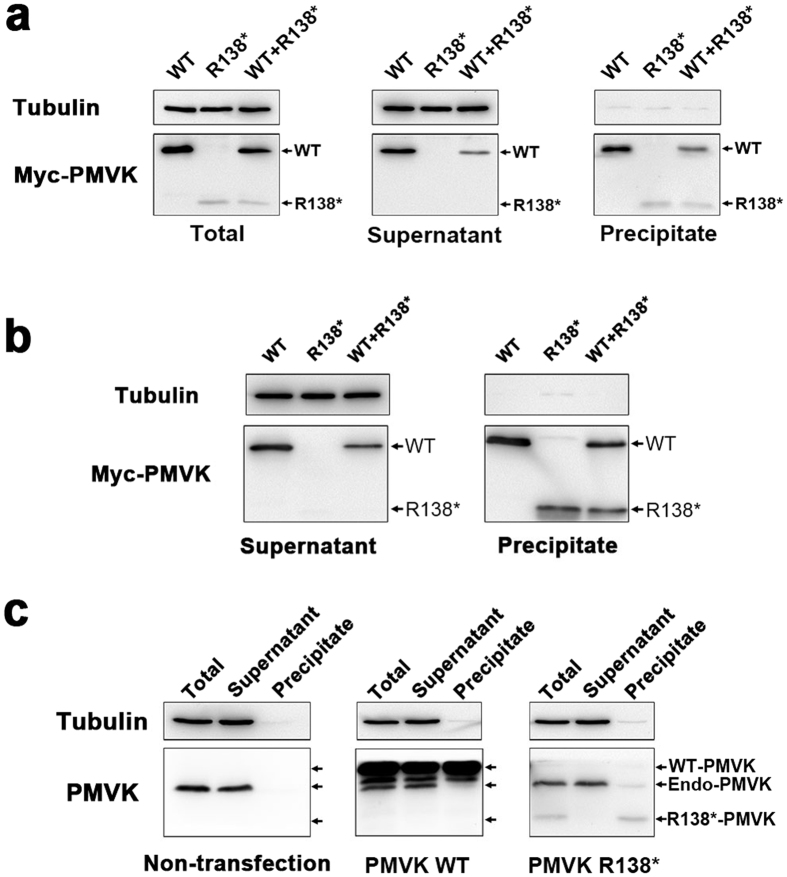
The Solubility of Endogenously and Exogenously Expressed WT and R138* PMVK in cells. (**a**) The R138* mutant PMVK shows reduced expression and solubility in HaCaT cells. Myc-tagged WT and R138* PMVK were expressed in HaCaT cells. The total level of WT PMVK was higher than that of mutant PMVK. WT PMVK was present in both soluble (supernatant) and insoluble (precipitate) fractions, but mutant PMVK was exclusively present in the precipitate. Tubulin was served as a marker for soluble proteins and also a loading control. (**b**) The R138* mutation disturbs the expression and solubility of PMVK in ARPE19 cells. Myc-tagged WT and R138* PMVK were exogenously expressed in ARPE19 cells. Soluble and insoluble fractions were separated and subjected to the detection of PMVK protein levels by Western blot. (**c**) The solubility of endogenous PMVK is not affected by exogenously expressed WT and R138* PMVK. In non-transfected HaCaT cells, PMVK was fully soluble. When HaCaT cells were transfected with Myc-tagged WT or R138* PMVK plasmids, endogenous PMVK was still largely present in the supernatant (soluble fraction). Tubulin was used as a loading control and a marker for soluble proteins.

To verify this finding, we transiently transfected HaCaT cells with Myc-tagged WT and mutant PMVK expression plasmids, and detected the distribution of PMVK in the supernatant (soluble) and the precipitated (insoluble) fractions of cell lysates using Western blot, respectively. As seen in Fig. 3a, the total expression level of WT PMVK was much higher than that of R138^*^ mutant PMVK driven by the CMV promoter, indicating that the mutation reduced the expression of PMVK. In addition, we found mutant PMVK was completely absent in the soluble fraction. In contrast, nearly half of the WT PMVK was present in the soluble fraction when overexpressed separately or together with mutant PMVK. Similar results were obtained in a non-skin-derived epithelial cell line ARPE-19 (Fig. 3b). These data demonstrated that the R138* mutation could disturb the expression and solubility of PMVK in cultured mammalian cells.

As a cytosolic enzyme, the solubility of PMVK is critical for its proper functioning. Therefore, the insoluble mutant PMVK is expected to be inactive. To further explore the properties of endogenous PMVK, we replicated the experiment in non-transfected and, WT or R138* PMVK-transfected HaCaT cells. We found that endogenous PMVK was fully soluble, and its solubility was not affected by exogenous expression of WT or mutant PMVK (Fig. 3c). This result indicated that the R138* mutation did not show a dominant negative effect on PMVK solubility. Based on this finding and our immunofluorescence data, we concluded that the R138* variation was a loss-of-function mutation which resulted in disturbed subcellular localization, expression, and stability of PMVK.

### Abnormal apoptosis and differentiation of keratinocytes in the lesional tissues of PMVK-deficient individuals

**Figure 4:**
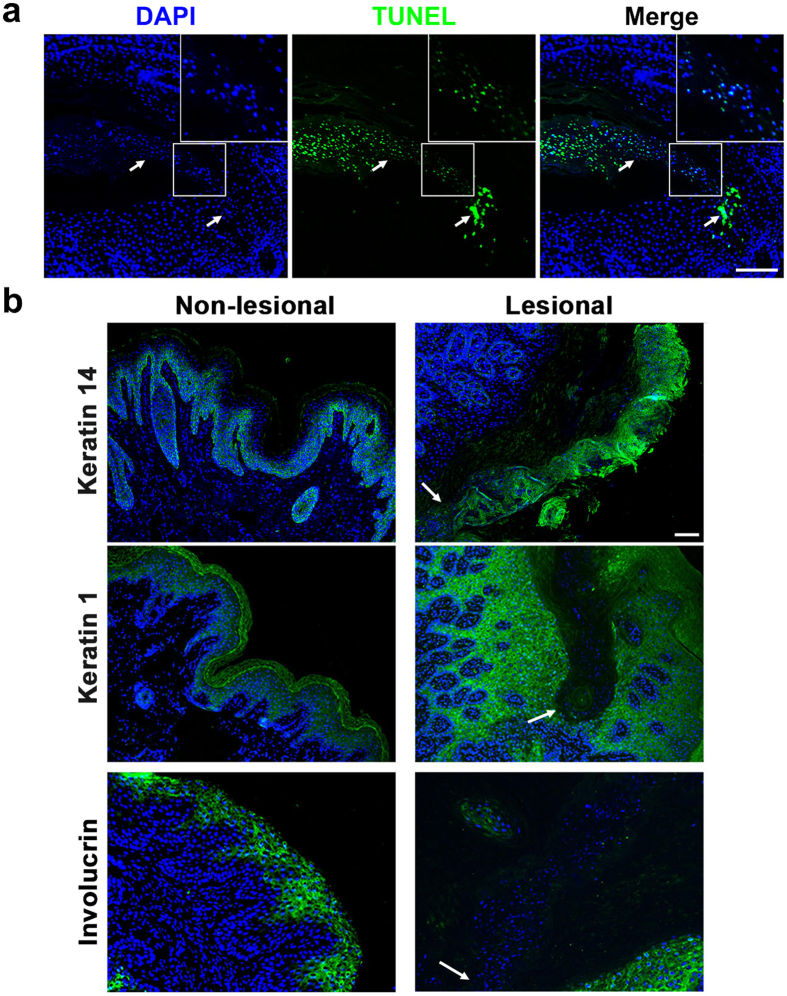
Abnormal Apoptosis and Differentiation of Keratinocytes in the Lesional Tissues of PMVK-deficient Individuals. (**a**) Apoptosis of keratinocytes in the lesional tissues. TUNEL-positive cells were observed in and under the cornoid lamella (indicated by arrows). The enlarged images show the co-localization of TUNEL signals and nuclei. Scale bar, 50 μm. (**b**) Aberrant distributions of the keratinocyte differentiation markers in the lesional tissues. Antibodies against keratin 14, keratin1or involucrin were used to reveal the differentiation process from immature stratum basale cells to mature keratinocytes in stratum granulosum and stratum corneum. The nuclei of keratinocytes in the lesional and non-lesional tissues were visualized by DAPI staining. The arrows indicate the bottom of cornoid lamellae. Scale bar, 20 μm.

Premature apoptosis and dysregulated keratinization of keratinocytes have been identified in several types of porokeratosis (such as PM, DSAP, and DSP) and are thought to be involved in the pathogenesis of porokeratosis^17^. To confirm this finding, we performed an *in situ* apoptosis assay in the lesional tissue of the proband in Family 1, using terminal deoxynucleotidyl transferase dUTP nick end labeling (TUNEL) technology. TUNEL-positive signals were observed in the cornoid lamella and the spinous layer exactly under the cornoid lamella (Fig. 4a).

Furthermore, we performed immunohistofluorescence analysis of lesional and non-lesional epidermis around the cornoid lamella from PMVK-deficient patients, using antibodies against keratin 14, keratin 1, or involucrin. Keratin 14 labels the immature keratinocytes which are located principally in the stratum basale of the epidermis. We observed the expression of keratin 14 in the cornoid lamella (Fig. 4b, upper panel), indicating that cells in the cornoid lamella were not fully differentiated. As a differentiation marker of keratinocytes in the spinous layer, keratin 1 is specifically expressed in the suprabasal cell layer of the epidermis. When compared to non-lesional tissues, keratin 1 was undetectable in the cornoid lamella and the granular layer, while the expression of keratin 1 in the spinous layer was unchanged (Fig. 4b, middle panel). Involucrin, a protein component of the epidermal barrier which is synthesized in the stratum spinosum and concentrated in the stratum granulosum, was also absent in the cornoid lamella and the granular layer (Fig. 4b, lower panel).

## Discussion

In this study, we investigated autosomal dominant porokeratosis in two large extended families and found that DSP affected individuals carried the *PMVK*c.412C > T (p.Arg138*) mutation. This mutation cosegregated with the DSP phenotype in both families. A recent study provided evidence that phosphomevalonate kinase (*PMVK*), mevalonate (diphospho) decarboxylase (*MVD*) and farnesyl diphosphate synthase (*FDPS*) are putative causal genes for porokeratosis^18^. It included, nine *PMVK*-deficient male patients affected with PM, HPM (disseminated superficial porokeratosis), genital porokeratosis, giant plaque of porokeratosisptychotropica (PPT), porokeratoma, or LP^18^. In our study, 23 patients in two five-generation families were diagnosed as having DSP in terms of the cutaneous and histopathological examination results and the associated clinical characteristics of the subjects (Table 1). Our genetic and functional study results showed that the loss of function mutation in *PMVK* led to autosomal dominant DSP.

Interestingly, all 23 affected members (11 females and 13 males) of these two large families carried the same c.412C > T (p.Arg138*) mutation in *PMVK*. Nevertheless, affected individuals showed substantial variation in clinical symptoms and severity of DSP. Even for identical twins in Family 1 (IV:9 and IV:12), they had differences in age of onset along with distribution and severity of their lesions (Table 1). Ultraviolet radiation is known to be an important trigger in the development of DSAP, yet none of the affected members in these two DSP families reported exacerbations after sun exposure. Different from those affected by DSAP, DSP patients in these two families experienced exacerbations in spring or autumn months. Patient IV:4 in Family 2 experienced exacerbations of DSP during pregnancy. The proband in Family 1 was the only vegetarian and had the most severe form of DSP. These findings suggest that the severity of PMVK deficiency-associated DSP is influenced by environmental factors such as diet but not by ultraviolet radiation. Among the 23 patients, two were found to have lesions located on their scrota, supporting the hypothesis that localized genital porokeratosis was the unique phenotype associated with *PMVK* mutations^18^. It is noteworthy that one patient with the *PMVK* mutation had hypertrophic plaques at the lesional regions (Figure S1d).

PMVK catalyzes the fifth reaction of the cholesterol/isoprenoid biosynthetic pathway^11^. To verify the effect of the *PMVK* mutation on the biological function of PMVK, we carried out a series of experiments outlined above. We noticed that this mutation abolished the co-localization of PMVK and MVK and dramatically reduced solubility of PMVK, leading to the loss of PMVK catalytic activity and the blockage of the mevalonate pathway. As a result, the synthesis of cholesterol and isoprenoid in HaCaT cells is impeded. By integrating our research results with previous findings^18^, we surmised that the mevalonate pathway might play an important role in maintaining the normal function of human epidermis, and inhibition of the mevalonate metabolism might be a driver of *MVK-* and *PMVK-*associated porokeratosis.

The end products of the mevalonate pathway, such as cholesterol and nonsterol isoprenoids, play important roles in multiple cellular processes including regulation of cell growth and differentiation^11^,^19^. Cholesterol is one of the three components of the extracellular lipid matrix in the stratum corneum (also known as skin barrier), and plays an essential role in the formation and functional maintenance of the skin barrier^20^,^21^. Cholesterol depletion has been reported to cause death of keratinocytes, neuronal cells, and tumor cells^22^,^23^,^24^. All these findings suggest that the cholesterol/isoprenoid biosynthetic pathway might be a crucial metabolic pathway in the skin. Considering the aberrant apoptosis and abnormal differentiation of keratinocytes in the PMVK-deficient lesional tissues, further studies will likely reveal the relationship between the cholesterol/isoprenoid biosynthetic pathway and the formation of cornoid lamellae in patients with PMVK-deficiency-associated DSP.

In summary, our study advances current knowledge in the field regarding the pathogenesis of DSP (a specific clinical subtype of porokeratosis) and the function of *PMVK*. It also helps elucidate the molecular mechanism of mutant *PMVK* associated DSP. Our findings suggest that the accumulation of abnormal metabolites or the shortage of cholesterol/isoprenoids may cause PMVK deficiency-associated porokeratosis. In addition, the mevalonate to cholesterol/isoprenoid biosynthetic pathway may be a potential target for treatment of idiopathic porokeratosis. The IRB number for this study is No. 201507002.

## Methods

### Informed consent

Our study was conducted according to the Declaration of Helsinki Principles. Informed consent was obtained from the participants, and the study protocol was approved by the ethical committee of College of Life Science and Technology, Huazhong University of Science and Technology, Wuhan, China.

### Exome capture, sequencing, and variation detection

Genomic DNA was isolated from peripheral venous blood samples of all available family members. We performed exome capture using the Agilent SureSelect Human All Exon Kit 51 m (v4) (Agilent Technologies) and massively parallel sequencing using the HiSeq2000 platform (Illumina) to generate an average of 4.11 billion bases of sequences as paired-end 90-bp reads.

After mapping to the human reference genome (hg19) with the BWA software, we obtained an average of 2.7 G bases (per sample) mapped to target exome regions with a mean depth of 52.5 times. On average, 96.97% of the exomes were covered at least 10 times.

The GATK program was applied to perform base quality score recalibration, indel realignment, duplicate removal, SNP and INDEL discovery, and genotype scoring using standard filtering parameters according to the GATK Best Practices recommendations^25^, and an average of 87,248 genetic variants were identified.

The identified variants were annotated by ANNOVAR^26^. We obtained 294, 317, or 308 new variation sites for each sample. Fifteen variants were shared by two affected individuals but absent in unaffected individuals.

### Construction of Expression Vectors

Site-directed mutagenesis was used to generate the R138* mutant form of human *PMVK*. Wild-type and R138* *PMVK* cDNA were cloned into plasmids pCMV-Myc (Clontech) and pGEX-4T (GE Healthcare) for expression in mammalian cells and *E. coli*. The full-length coding sequence of MVK was cloned into the p3XFLAG-CMV-7.1 (MBL) mammalian expression vector. All constructs were verified by Sanger sequencing.

### Separation of soluble and insoluble components in HaCaT cells

HaCaT cells were transiently transfected with Myc-tagged WT or R138* mutant PMVK or both. After 48 hours, the cells were harvested and lysed with immunoprecipitation lysis buffer, followed by sonication of total protein extractions. To separate soluble and insoluble proteins, cell lysates were centrifuged at 13,000 rpm for 20 minutes at 4°C. The supernatant was collected as the soluble component and the precipitate was washed once with phosphate buffered saline (PBS) and kept as the insoluble component.

### Western blot

Human HaCaT Cells were lysed in immunoprecipitation lysis buffer containing a mixture of protease inhibitors (0.25 mM PMSF, 10 mg/ml aprotinin and leupeptin, and 1 mM DTT). 10 ug of total protein was separated *via* 12% SDS-PAGE and transferred to a nitrocellulose membrane. The membranes were probed with antibodies against Tubulin, Myc (Proteintech) or PMVK (Proteintech).

### TUNEL and immunohistofluorescence analyses

The TUNEL reaction was performed as described previously^17^. The biospecimen of PMVK-deficient lesions (from the proband in Family 1) was stained with antibodies against keratin 14, keratin1, or involucrin (Proteintech) and then with the secondary antibody, i.e., the Alexa Fluor 488 goat anti-rabbit immunoglobilin G (Life Technologies). The immunohistofluorescence analysis was performed as reported previously^27^.

### Confocal Microscopy

HaCaT cells were fixed in 4% paraformaldehyde for 30 min after being transiently transfected with Myc-tagged WT or R138* mutant PMVK expression vector together with Flag-tagged MVK expression vector for 48 hours. Then, they were washed three times with PBS. Cells were visualized by a confocal microscope (Olympus).

### Informed consent

All the authors and patients reviewed the manuscript and approved the submission.

## Additional Information

**How to cite this article**: Wang, J. *et al*. Loss-of-function Mutation in *PMVK* Causes Autosomal Dominant Disseminated Superficial Porokeratosis. *Sci. Rep.* **6**, 24226; doi: 10.1038/srep24226 (2016).

## Supplementary Material

Supplementary Information

## Figures and Tables

**Table 1:** Clinical Data of the Patients in the Two DSP Families.

Family	Subject	Gender	Age of onset (years)	Affected Regions
Family 1	II:6	Female	18	Forehead, trunk, limbs, feet
	III:3	Male	15	Forehead, neck, trunk, limbs, feet, groin, scrotum, perianal. region, buttock
	III:11	Female	16	Forearm
	III:13	Female	20	Toes
	III:16	Male	18	Forehead, limbs
	III:18	Female	16	Hands
	IV:1	Male	20	Limbs, perianal region, groin, feet
	IV:4	Female	17	Forearm
	IV:9	Male	29	Neck, groin, feet
	IV:12	Male	35	Limbs
	IV:15	Male	26	Groin
	IV:17	Male	15	Scrotum
Family 2	II:2	Female	5~10^1^	Toes, fingers
	II:5	Male	5~10^1^	Neck, lower limbs
	II:7	Female	5~10^1^	Feet, fingers
	II:9	Female	5~10^1^	Fingers, buttock
	III:7	Male	5~10^1^	Forearm, lower limbs
	III:9	Female	5~10^1^	Toes, fingers
	III:10	Male	5~10^1^	Head, neck, back, limbs
	III:16	Male	5~10^1^	Feet, fingers
	IV:3	Female	5~10^1^	Toes, fingers
	IV:4	Female	5~10^1^^,^^2^	Trunk, limbs, palms
	V:1	Male	10	Feet, fingers

^1^The age of onset were mostly from 5 to 10 years old according to the patients’memories. The symptoms aggravated around their 16 years old, usually showed a recurrent episodes pattern.

^2^IV:4 showed hypertrophic plaquesat the lesional regions of the forearm and palm.

## References

[b1] Sertznig P., von Felbert V. & Megahed M. Porokeratosis: present concepts. J Eur Acad Dermatol 26, 404–412 (2012). 10.1111/j.1468-3083.2011.04275.x 21929548

[b2] Online Mendelian Inheritance in Man, OMIM^®^. McKusick-Nathans Institute of Genetic Medicine, Johns Hopkins University (Baltimore, MD). Available at: http://www.omim.org/. (Accessed: 8th September 2015).

[b3] Zhang S. . Exome sequencing identifies MVK mutations in disseminated superficial actinic porokeratosis. Nat Genet 44, 1156–1160 (2012). 22983302 10.1038/ng.2409

[b4] Cui H. . Exome sequencing identifies SLC17A9 pathogenic gene in two Chinese pedigrees with disseminated superficial actinic porokeratosis. J Med Genet 51, 699–704 (2014). 25180256 10.1136/jmedgenet-2014-102486

[b5] Zeng K., Zhang Q., Li L., Duan Y. & Liang Y. Splicing mutation in MVK is a cause of porokeratosis of Mibelli. Arch Dermatol Res 306, 749–755 (2014). 24781643 10.1007/s00403-014-1465-7

[b6] Cao H. M. . Identification of a locus (DSP2) for disseminated superficial porokeratosis at chromosome 12q21.2-24.21. Clin Exp Dermatol 37, 672–676 (2012). 22680787 10.1111/j.1365-2230.2012.04380.x

[b7] Wei S. . A Novel Locus for Disseminated Superficial Porokeratosis Maps to Chromosome 18p11.3. J. Invest. Dermatol 123, 872–875 (2004). 15482473 10.1111/j.0022-202X.2004.23455.x

[b8] McKenna A. . The Genome Analysis Toolkit: A MapReduce framework for analyzing next-generation DNA sequencing data. Genome Res 20, 1297–1303 (2010). Available at: https://www.broadinstitute.org/gatk/. (Accessed: 9th July 2014). 20644199 10.1101/gr.107524.110 PMC2928508

[b9] Database of Single Nucleotide Polymorphisms (dbSNP). Bethesda (MD): National Center for Biotechnology Information, National Library of Medicine. (dbSNP Build ID: {137}). Available at: http://www.ncbi.nlm.nih.gov/SNP/. (Accessed: 5th October 2014).

[b10] McVean . An integrated map of genetic variation from 1,092 human genomes. Nature. (2012) Available at: http://browser.1000genomes.org. (Accessed: 5th October 2014). 10.1038/nature11632 PMC3498066 23128226

[b11] Rauthan M. & Pilon M. The mevalonate pathway in C. elegans. Lipids Health Dis 10, 243 (2011). 22204706 10.1186/1476-511X-10-243 PMC3274489

[b12] Chambliss K. L., Slaughter C. A., Schreiner R., Hoffmann G. F. & Gibson K. M. Molecular cloning of human phosphomevalonate kinase and identification of a consensus peroxisomal targeting sequence. J Biol Chem 271, 17330–17334 (1996). 8663599 10.1074/jbc.271.29.17330

[b13] Olivier L. M., Chambliss K. L., Gibson K. M. & Krisans S. K. Characterization of phosphomevalonate kinase: chromosomal localization, regulation, and subcellular targeting. J Lipid Res 40, 672–679 (1999). 10191291

[b14] Hogenboom S. . Human mevalonate pyrophosphate decarboxylase is localized in the cytosol. Mol Genet Metab 81, 216–224 (2004). 14972328 10.1016/j.ymgme.2003.12.001

[b15] Herdendorf T. J. & Miziorko H. M. Functional Evaluation of Conserved Basic Residues in Human Phosphomevalonate Kinase. Biochemistry 46, 11780–11788 (2007). 17902708 10.1021/bi701408t PMC2530820

[b16] Pilloff D. . The Kinetic Mechanism of Phosphomevalonate Kinase. J Biol Chem 278, 4510–4515 (2003). 12424232 10.1074/jbc.M210551200

[b17] Shen C. S. . Premature apoptosis of keratinocytes and the dysregulation of keratinization in porokeratosis. Br. J. Dermatol 147, 498–502 (2002). 12207590 10.1046/j.1365-2133.2002.04853.x

[b18] Zhang Z. . Genomic variations of the mevalonate pathway in porokeratosis. Elife 4, e06322 (2015). 26202976 10.7554/eLife.06322 PMC4511816

[b19] Buhaescu I. & Izzedine H. Mevalonate pathway: A review of clinical and therapeutical implications. Clin Biochem 40, 575–84 (2007). 17467679 10.1016/j.clinbiochem.2007.03.016

[b20] Bouwstra J. A. & Ponec M. The skin barrier in healthy and diseased state. Biochim Biophys Acta 1758, 2080–2095 (2006). 16945325 10.1016/j.bbamem.2006.06.021

[b21] Iwai I. . The human skin barrier is organized as stacked bilayers of fully extended ceramides with cholesterol molecules associated with the ceramide sphingoid moiety. J. Invest. Dermatol 132, 2215–2225 (2012). 22534876 10.1038/jid.2012.43

[b22] Michikawa M. & Yanagisawa K. Inhibition of Cholesterol Production but Not of Nonsterol Isoprenoid Products Induces Neuronal Cell Death. J. Neurochem 72, 2278–2285 (1999). 10349836 10.1046/j.1471-4159.1999.0722278.x

[b23] Calay D. . Inhibition of Akt Signaling by Exclusion from Lipid Rafts in Normal and Transformed Epidermal Keratinocytes. J. Invest. Dermatol 130, 1136–1145 (2010). 20054340 10.1038/jid.2009.415

[b24] Mohammad N. . Cholesterol depletion by methyl-β-cyclodextrin augments tamoxifen induced cell death by enhancing its uptake in melanoma. Mol. Cancer 13, 204 (2014). 25178635 10.1186/1476-4598-13-204 PMC4175626

[b25] DePristo M. A. . A framework for variation discovery and genotyping using next-generation DNA sequencing data. Nat Genet 43, 491–498 (2011). 21478889 10.1038/ng.806 PMC3083463

[b26] Wang K., Li M. & Hakonarson H. ANNOVAR: functional annotation of genetic variants from high-throughput sequencing data. Nucleic Acids Res 38, e164 (2010). 20601685 10.1093/nar/gkq603 PMC2938201

[b27] Zhang C. . Mutations in ABCB6 Cause Dyschromatosis Universalis Hereditaria. J Invest Dermatol 133, 2221–2228 (2013). 23519333 10.1038/jid.2013.145

