# Supplementary material for: Loss-of-function Mutation in PMVK Causes Autosomal Dominant Disseminated Superficial Porokeratosis
Source: Sci Rep. 2016 Apr 7;6:24226. doi: 10.1038/srep24226 (PMC4823745; doi:10.1038/srep24226)
Supplement: Supplementary Information [file srep24226-s1.pdf]

Supplementary Data

Loss-of-function Mutation in *PMVK* Causes Autosomal Dominant Disseminated Superficial Porokeratosis

Jiuxiang Wang[1,+], Ying Liu[1,+], Fei Liu[1,+],Changzheng Huang[2], Shanshan Han[1], Yuexia Lv[1], Chun-Jie Liu[3], Su Zhang[4], Yayun Qin[1], Lei Ling[5], Meng Gao[1], Shanshan Yu[1], Chang Li[1], Mi Huang[1], Shengjie Liao[1], Xuebin Hu[1], Zhaojing Lu[1], Xiliang Liu[1], Tao Jiang[1], Zhaohui Tang[1], Huiping Zhang[6], An-Yuan Guo[3,*], Mugen Liu[1,*]

Supplementary Figures

Supplementary Figure1. Pedigree, Clinical Phenotype and Mutation information of Family2 with DSP

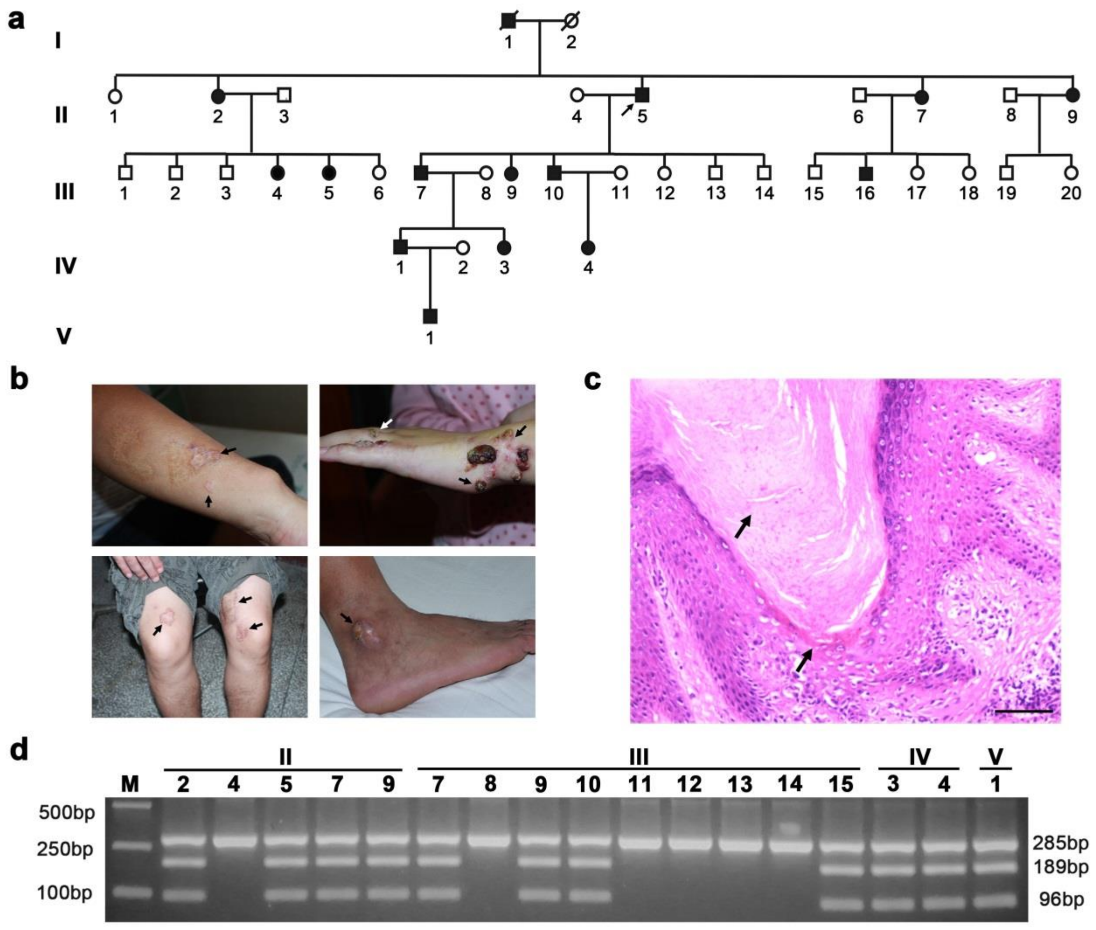

(a) Pedigree of Family2. Black symbols, affected individuals; White symbols, unaffected individuals; Arrow, the proband of the Family.

(b) Typical lesions are distributed on forearms, hands, legs and feet of the patients in Family2 (indicated by arrows).

(c) Histological examination of the keratotic lesions in the proband. The arrows indicated the huge cornoid lamella with decreased granular cells below and around the parakeratotic column. Scale bar, 25 μm.

(d) Segregation analysis of the c.412C>T mutation in Family2. The restriction fragment length polymorphism (RFLP) analysis showed complete segregation of the mutation with the DSP phenotype in Family2.

Supplementary Figure 2. Haplotype Analysis for Six SNPs Surrounding *PMVK* in Family1

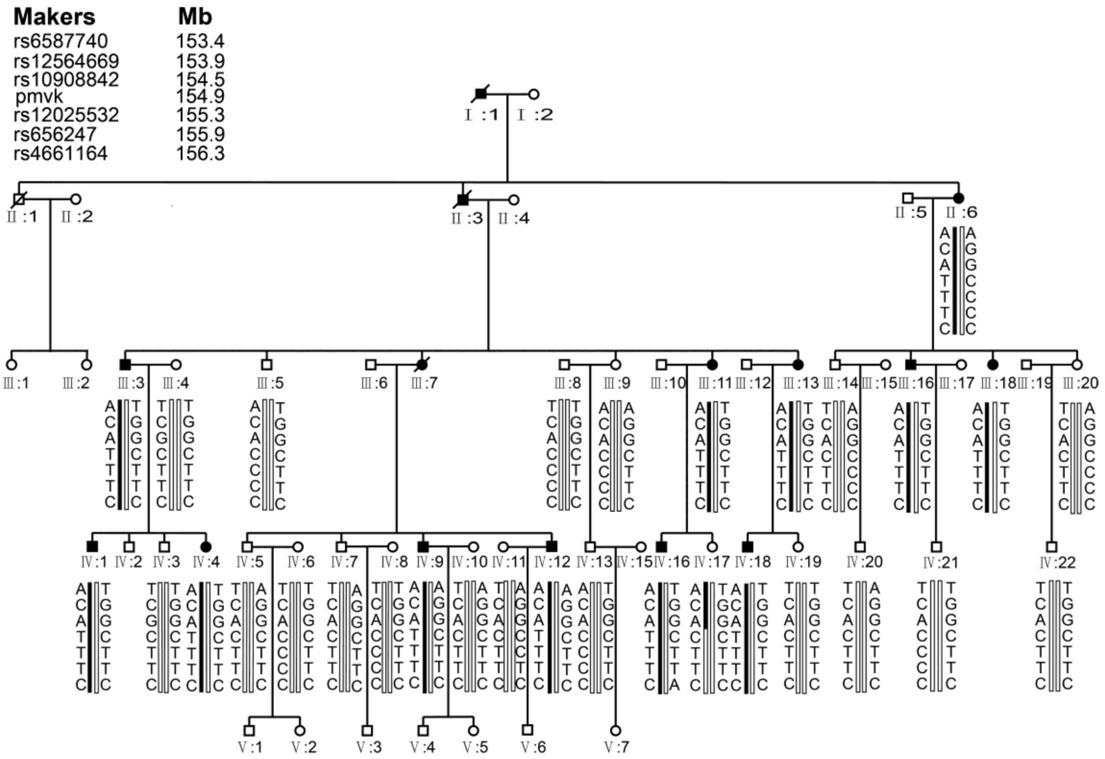

Black symbols, affected individuals; White symbols, unaffected individuals. SNP markers are listed from top to bottom followed by their chromosomal positions: rs6587740, rs12564669, rs10908842, rs12025532, rs656247, rs4661164. The disease-causing haplotypes are indicated by vertical black bars, except for IV:17. There is a DNA exchange happened between rs10908842 and *PMVK*.

Figure S3.Prokaryotic expression of GST-tagged WT and R138* PMVK.

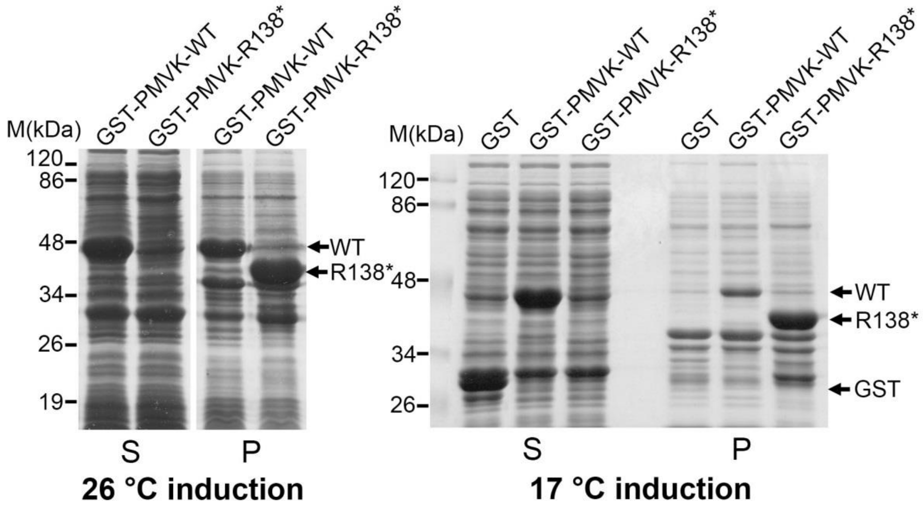

The arrows indicated the WT and mutant GST-tagged PMVK, respectively, detected by coomassie blue staining. The mutant PMVK formed inclusion bodies under both induction temperatures, whereas the WT PMVK is almost soluble under the same condition. S, supernatant; P, precipitate.

Supplementary Tables

Supplementary Table 1. Bioinformatics Analysis of the Whole-exome Sequencing Data

	IV:1 (affected)	II:6 (affected)	III:14 (normal)
Number of raw bases (Mb)	4244	3732	4356
Number of bases mapped to target region (Mb)	2729	2653	2711
Mean depth of target region	53.11	51.63	52.77
Coverage of target region (%)	99.69	99.48	99.64
Coverage of target region with at least 10x (%)	97.05	96.43	96.79
Coverage of target region with at least 20x (%)	89.46	88.82	89.13
Total variants	76429	75502	78350
Nonsynonymous, splicing and InDel variants	18244	17711	18452
Above variants with MAF[a]<0.05	9223	9232	9222
Above variants absent in the dbSNP137 database	294	317	308
Above variants shared by the patients but absent in the unaffected individual	19		
Above variants confirmed by Sanger sequencing and segregation test	1 [*PMVK*, c.412C>T (p.Arg138*)]		

[a] MAF, minor allele frequency

Supplementary Table 2. Primers Used in the Study		
	**Name**	**Sequence(5'-3')**
Primers for *PMVK* mutation screening	Exon1 F	ggaaatttcagacgtcaccc
	Exon1 R	aaagctcactccgtgacacc
	Exon2 F	aggcctcttgactccctttc
	Exon2 R	ggaatcatgccagtgactcag
	Exon3 F	tggctcttccgctgttttag
	Exon3 R	atggtggcagtggagacag
	Exon4 F	ctgccctgggttctgtagtc
	Exon4 R	aaaccacagggtgggtagc
	Exon5 F	actccagttggggttagcag
	Exon5 R	catgacaccctaacgccttc
Primers for RFLP[a] analysis of the c.412C>T mutation of *PMVK*	Alu I-F	ggtgagtgacacacggagagtg
	Alu I-R	tgaccctcaggattctgtcc
Primers for constructing the expression plasmids of *PMVK* and *MVK*	pCMV-Myc-PMVK-F	gcccGAATTCggatggccccgctgggaggc
	pCMV-Myc-PMVK-R	ccgcGGTACCctaaagtctggagcggataa
	pGEX-4T-PMVK-F	ccgGAATTCatggccccgctgggag
	pGEX-4T-PMVK-R	ccgCTCGAGctaaagtctggagcgg
	pCMV-Flag-MVK-F	ccgcGAATTCaatgttgtcagaagtcctactggtgtctg
	pCMV-Flag-MVK-R	cccgGGATCCtcagaggccatccagggctt

[a]RFLP, restriction fragment length polymorphism
